# Supplementary material for: Evidence supported by Mendelian randomization: impact on inflammatory factors in knee osteoarthritis
Source: Front Med (Lausanne). 2024 May 28;11:1382836. doi: 10.3389/fmed.2024.1382836 (PMC11165061; doi:10.3389/fmed.2024.1382836)
Supplement: Supplementary file 1 [file Data_Sheet_1.ZIP › data/GCST90274834_5e6/834.leaveoneout.pdf]

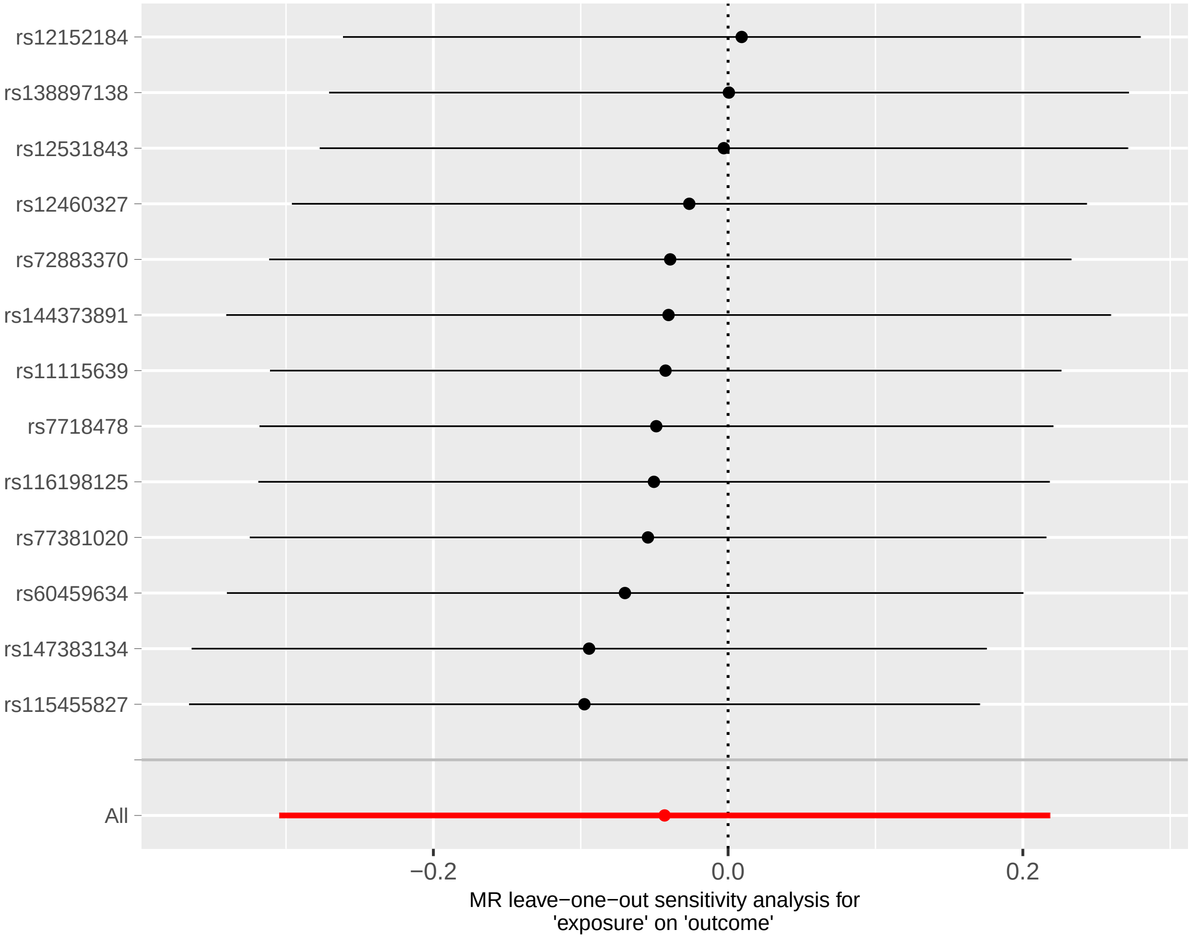
rs12152184
rs138897138
rs12531843
rs12460327
rs72883370
rs144373891
rs11115639
rs7718478
rs116198125
rs77381020
rs60459634
rs147383134
rs115455827
All
−0.2
0.0
0.2
MR leave−one−out sensitivity analysis for
'exposure' on 'outcome'
